# The Limited Palette for Photonic Block‐Copolymer Materials: A Historical Problem or a Practical Limitation?

**DOI:** 10.1002/ange.202117275

**Published:** 2022-04-21

**Authors:** Zhen Wang, Chun Lam Clement Chan, Richard M. Parker, Silvia Vignolini

**Affiliations:** ^1^ Yusuf Hamied Department of Chemistry University of Cambridge Cambridge CB2 1EW UK

**Keywords:** Block-Copolymers, Mesophases, Nanostructures, Photonic Crystals, Self-Assembly

## Abstract

Block‐copolymer self‐assembly has proven to be an effective route for the fabrication of photonic films and, more recently, photonic pigments. However, despite extensive research on this topic over the past two decades, the palette of monomers and polymers employed to produce such structurally colored materials has remained surprisingly limited. In this Scientific Perspective, the commonly used block‐copolymer systems reported in the literature are summarized (considering both linear and brush architectures) and their use is rationalized from the point of view of both their historical development and physicochemical constraints. Finally, the current challenges facing the field are discussed and promising new areas of research are highlighted to inspire the community to pursue new directions.

## Graphical abstract

The self‐assembly of block‐copolymers into photonic films and pigments has drawn significant attention due to the tunability of the optical response. In this Scientific Perspective we identify and rationalize the limited range of monomers and polymerization methods employed to produce such structurally colored materials to date. By considering historical development and practical limitations, new strategies are proposed to inspire new research directions.

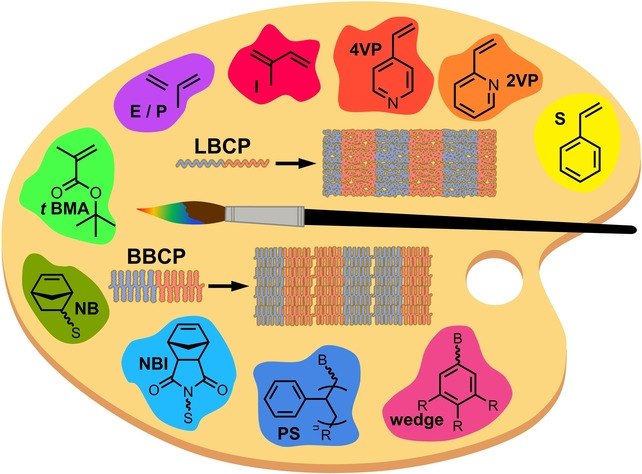

## 1 Introduction

As a bottom‐up method, block‐copolymer (BCP) self‐assembly has proven to be useful for the design and preparation of a diverse library of nanostructured materials, arising from the tunability of their molecular composition and an inherent capability to self‐organize into complex nanoscale architectures.^[1]^ Moreover, by matching the dimensions of such nanoarchitectures to the wavelengths of visible light, it is possible to fabricate photonic films that reflect vibrant, iridescent color.^[2]^ Finally, recent studies have shown that by geometrically confining the self‐assembly process, hierarchical photonic particles (referred to here as “photonic pigments”) can be produced that offer novel optical behavior.^[3]^

**Figure 1:**
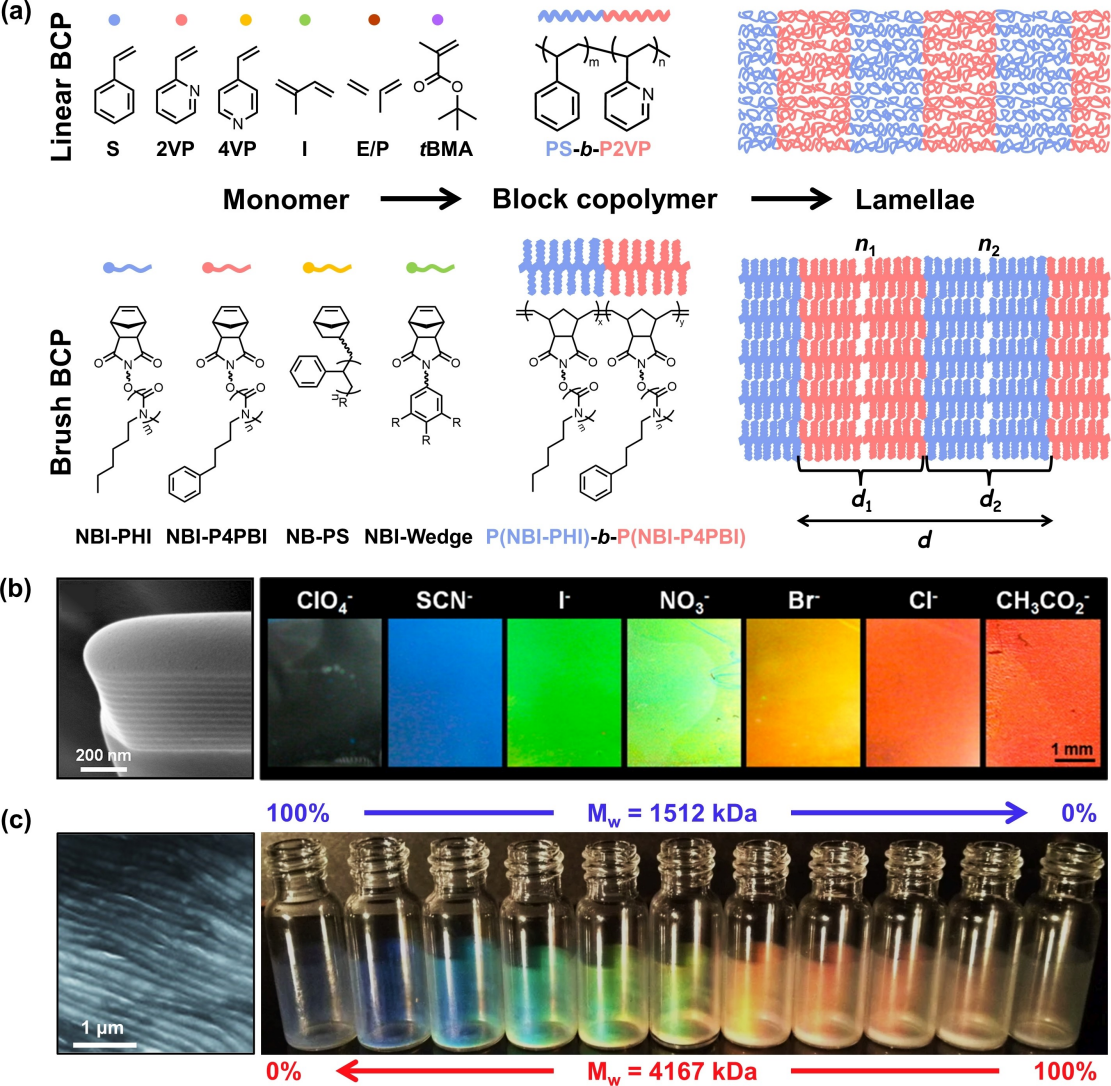
a) Chemical structures of common monomers used to produce linear and brush block‐copolymers (LBCPs and BBCPs, respectively) and a schematic showing their self‐assembly into lamellar structures. b) Scanning electron microscopy (SEM) image of a LBCP lamellar structure formed from PS‐*b*‐P2VP (left) and photographs showing the range of colored films that can be produced using counterion exchange to selectively swell the quaternized P2VP domains (right). Reproduced with permission from ref. [13] Copyright 2012 American Chemical Society. c) SEM image of a BBCP lamellar structure self‐assembled from P(I‐PHI)‐*b‐*P(I−P4PBI) with weight average molecular weight *M*_w_=1.5 MDa (left) and the range of colors that can be achieved by systematically blending into P(I‐PHI)‐*b*‐P(I−P4PBI) with *M*_w_=4.2 MDa (right). Adapted with permission from ref. [14] Copyright 2012 Wiley‐VCH.

A wide variety of photonic architectures have been self‐assembled from BCPs, ranging from long‐range ordered structures (e.g., closely packed micelles,^[4]^ hexagonally packed cylinders,^[5]^ double diamond,^[6]^ gyroids,^[7]^ cubic and correlated networks^[8]^), to systems where only short‐range order is present, such as photonic glasses.^[9]^ However, over the past two decades the majority of studies have focused upon the construction of lamellar structures from linear and brush block‐copolymers (respectively LBCPs and BBCPs), as illustrated in Figure 1. This nanoarchitecture is favored as it is both simple to obtain and it functions as a one‐dimensional photonic multilayer, which offers the best optical performance (i.e., maximal reflectivity from the smallest size). While previous reviews have summarized fabrication strategies and benchmarked optical performance,[^2^, ^10^] an overview is absent in this field from the point of view of the library of polymers employed. In this Scientific Perspective, we categorize and systemically analyze the BCPs used to date for photonic multilayer films and particles and, by highlighting the current challenges and limitations from a materials perspective, we propose new areas of opportunity that can be used to inform and direct future studies.

## 2 Classification of BCPs used for photonic materials

**Figure 2:**
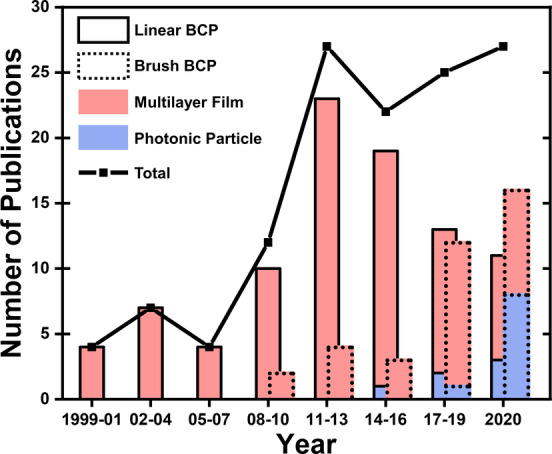
Line chart showing the number of articles published on this topic over the last 22 years, split into intervals of 3 years. The bar chart subdivides this research output into copolymer type (linear BCP vs brush BCP) and geometry (multilayer film vs photonic particle).

The concept of processing LBCPs into photonic films was first proposed in 1999,^[11]^ while the development of the *grafting‐through* approach via ring‐opening metathesis polymerization (ROMP) catalyzed the development of BBCPs for photonics a decade later.^[12]^ As summarized in Figure 2, the number of articles per year in the field of BCP photonics has grown steadily (black line); however, since 2016 there has been a clear transition from LBCP‐ to BBCP‐based systems (solid vs dashed bars). Moreover, while earlier studies focused on the development and characterization of new copolymer systems that could produce lamellar architectures, recent articles have instead tended towards increasing complexity or adding functionality, with a greater focus upon the application. The trend is particularly apparent by the growth of studies exploring confined self‐assembly within an emulsified droplet to produce photonic microparticles (red vs blue bars). This strategy offers both interesting optics (e.g., suppressed iridescence), and a scalable fabrication pathway towards photonic pigments, which have potential as an alternative to, for example, mica‐based effect pigments.^[3a]^

**Figure 3:**
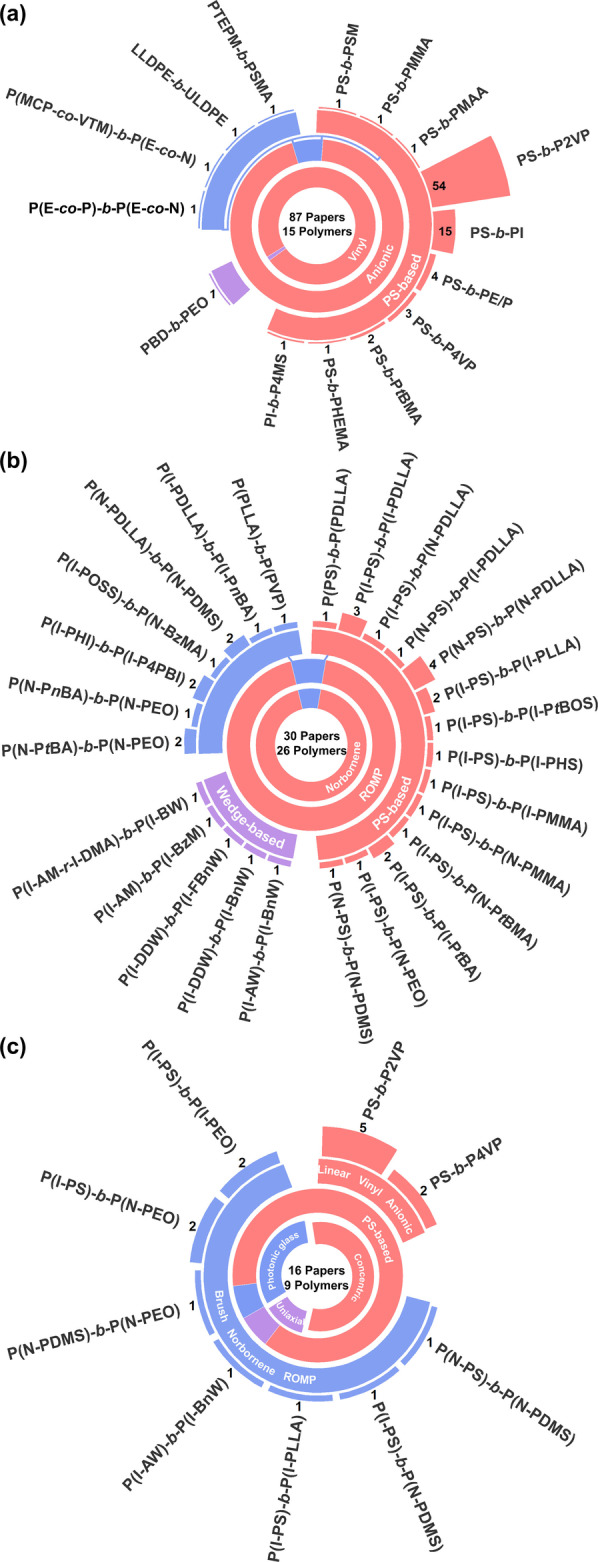
Nested pie charts summarizing the composition, monomer type, and polymerization method for a) photonic LBCP multilayer films, b) photonic BBCP multilayer films, and c) photonic microparticles prepared from both LBCPs and BBCPs. The outermost layer reports the name of the BCP and the number of papers that employ this system. The inner rings highlight the dominant polymerization method (e.g., ROMP or anionic polymerization) and monomer types (e.g., vinyl, norbornene). Note that in (c) “concentric”, “uniaxial”, and “photonic glass” represent the optical nanostructure of the particle and respectively correspond to a concentric or uniaxial multilayer geometry, or an inverse photonic glass.

To better comprehend the different monomers and polymerization strategies employed in the production of BCP‐based photonic materials, a database of all reported copolymer systems has been constructed; this is visualized in Figure 3, where the different systems are categorized by copolymer name (e.g., PS‐*b*‐P2VP), monomer type (e.g., styrene), polymerization method (e.g., ROMP), polymer architecture (e.g., linear vs brush BCP), and also grouped into polymer families (e.g., “PS‐based” for styrene or styrene derivatives) and assembly geometry (e.g., film vs particle). This analysis revealed that the most frequently used LBCP is by far PS‐*b*‐P2VP, which is found in 54 of the 87 articles that report photonic LBCP multilayer films and five of the seven articles reporting photonic LBCP particles (outer rings of Figure 3a and Figure 3c, respectively), which overall corresponds to 63 % of all reported LBCP systems. In comparison, the next most common combination, PS‐*b*‐PI, only accounts for 16 %. These statistics reveal that most studies have focused on improving the fabrication of PS‐*b*‐P2VP photonic multilayers or adding functionality, rather than exploring new monomer combinations, with only 13 other LBCPs having ever been reported in this context. Finally, anionic polymerization of vinyl monomers is the dominant synthetic pathway for LBCPs, accounting for 95 % of the database.

In the case of BBCPs, the majority of polymers have been synthesized from norbornene‐based macromonomers (MMs) via ROMP (95 %). Analogous to the LBCP literature, PS is used as a side chain in a large proportion of the MM combinations (accounting for 59 % of BBCP publications). However, the unique geometry of BBCPs also gives rise to other types of monomers not available to LBCPs. Notably, wedge‐based MMs have been employed in a significant portion of articles studying photonic BBCPs (14 %). The dendronized nature of wedge‐based MMs makes them particularly useful in BBCP systems due to their much higher steric congestion compared to polymer side chains of the same molecular weight (MW). This effectively increases the rigidity of the BBCP without sacrificing access to the high MWs required for direct self‐assembly into photonic structures.

**Figure 4:**
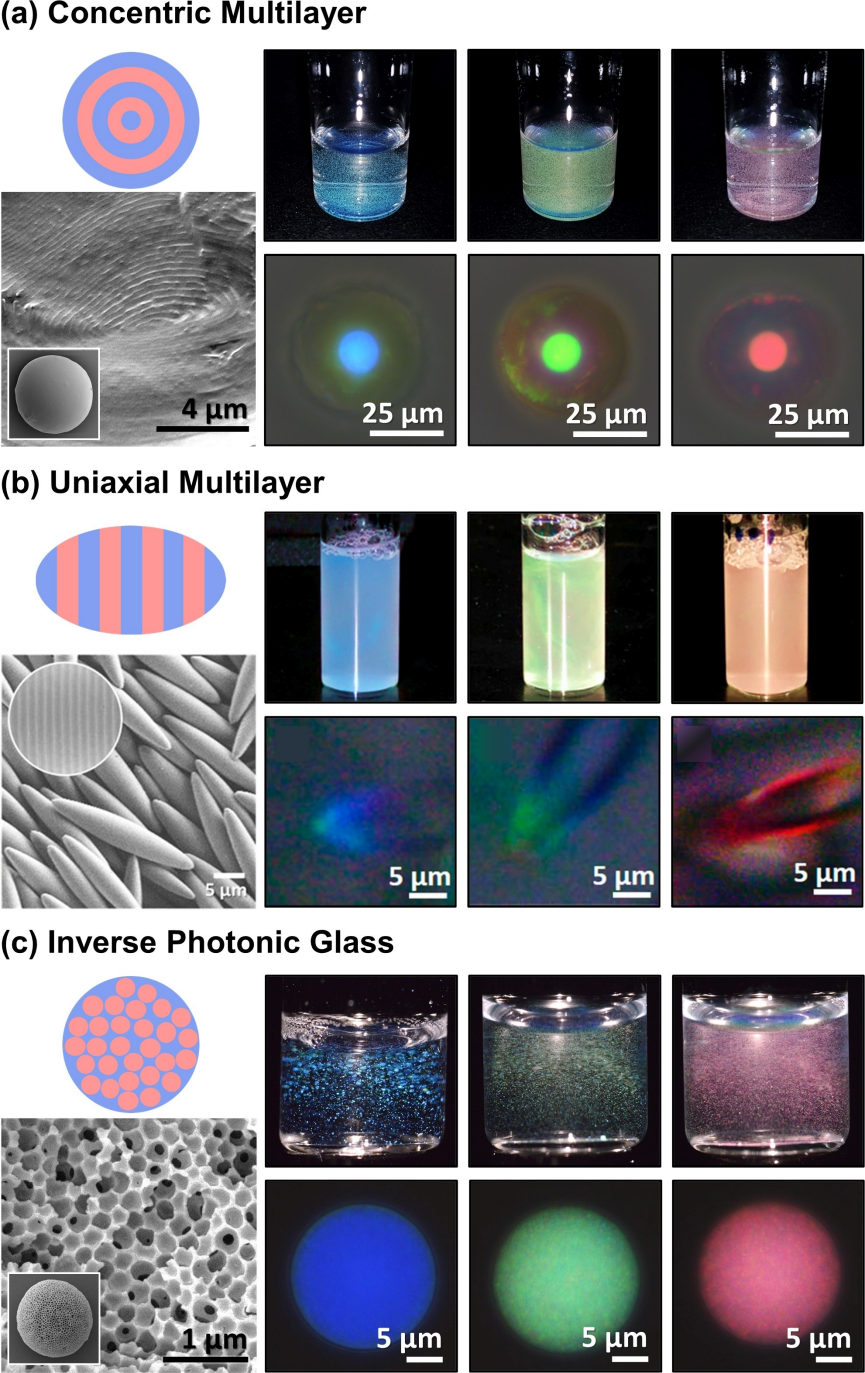
Examples of the three photonic BCP particle morphologies reported to date: a) concentric multilayer; adapted with permission.^[3a]^ Copyright 2019 American Chemical Society. b) Uniaxial multilayer; adapted with permission.^[3c]^ Copyright 2019 American Chemical Society. c) Inverse photonic glass; adapted under the terms of the CC‐BY license.^[3b]^ Copyright 2020 Wiley‐VCH. For each system (from left to right) a schematic of a particle is provided, along with an example SEM image showing the particle and its internal microstructure, and macroscopic and microscopic images of blue, green, and red particles.

The distributions remain similar when only considering BCPs assembled in confinement (Figure 3c). While it is unsurprising that researchers have initially applied the same limited set of copolymer systems to this nascent field, several trends have emerged regarding the relative efficacy of these polymers (Supporting Information, Tables S6, S8, and S9). Notably, while particles based on LBCPs typically require swelling to display visible coloration, which is often transient and solvent specific, the larger MWs and increased backbone rigidity achievable with BBCPs enables the direct production of visibly colored pigments.[^3a^, ^3c^, ^15^] Finally, by optimizing the BCP chemistry and particle fabrication, photonic particles with three distinct nanostructure geometries have been reported, as exemplified in Figure 4.^[2a]^

## 3 Historical and practical considerations on the photonic BCP library

Given the number of new polymers being synthesized each year, accelerated by the development of controlled radical polymerization methods (such as atom transfer radical polymerization (ATRP) and reversible addition‐fragmentation chain‐transfer polymerization (RAFT)), the limited diversity in terms of polymer composition reported in Figure 3 is surprising. Indeed, only 44 distinct BCPs have been developed for photonic structures over the past two decades. This is especially restricted for LBCPs, where only 15 different compositions have been reported to form structural color. The relationship between the composition of these LBCPs and the corresponding structural and optical properties of the assembled photonic materials are reported in Tables S5–S9. For simplicity, the nanostructure is quantified in terms of the photonic dimension (i.e., the lamellar domain spacing (*d*) or center‐to‐center correlation distance, (2*ξ*) for amorphous structures),^[16]^ while the characteristic wavelength of the reflectance peak (*λ*_max_) is used to describe the optical behavior.

### 3.1 The dominance of PS‐*b*‐P2VP

Considering the library of LBCPs in terms of their synthesis, it is striking that 93 % were produced by anionic polymerization of styrene monomers. This method has been historically favored as it is known to produce well‐defined LBCPs with high monomer conversion, with the potential for a large degree of polymerization (DP) and a low polydispersity index (i.e., typically PDI<1.10, Table S5).^[17]^ However, it is important to note that, rather than PDI, the determinant parameter for well‐formed lamellae is the block ratio, which needs to be symmetric to ensure a planar interface between domains.^[18]^ As such, while radical polymerization techniques typically result in a higher PDI, they retain sufficient control of the block ratio to produce photonic BCP materials,^[19]^ thus, potentially offering a simpler and more cost‐effective pathway.

The restricted monomer choice likely originates from the developmental process of anionic polymerization. While it is now used to synthesize polymers from a myriad of diverse monomers,^[20]^ early studies mainly focused on diene and aromatic molecules.^[21]^ As a result, the polymerization techniques and protocols for monomers, such as isoprene, styrene, and vinyl pyridine, are the most established. These “traditional” LBCPs include PS‐*b*‐P2VP, PS‐*b*‐PI, PS‐*b*‐PE/P, and PS‐*b*‐P4VP, which together account for 88 % of all studies summarized here. Notably, for the 27 different compositions of PS‐*b*‐P2VP, this robust approach has allowed for the number average molecular weight, *M*_n_, to range from 78 to 443 kDa with a block ratio (${\mathrm{DP}}_{\mathrm{PS}}/{\mathrm{DP}}_{P2\mathrm{VP}})$ of 0.9–1.4. Finally, it is important to note that many of these copolymers are also now commercially available, making them attractive and accessible starting materials from which to develop photonic systems. However, while the democratization of access to suitable polymers has been beneficial for the field, with >50 % of papers utilizing a commercial LBCP, it seems to have also stifled the development of new polymerization routes and limited the diversity of the polymers explored.

To understand why PS‐*b‐*P2VP has been so widely utilized, it is first necessary to consider the polymer traits that are desirable for a photonic multilayer film. For symmetrical lamellae (i.e., *d*_1_≅*d*_2_), the key parameters dictating the characteristic color of the photonic multilayer film are the domain spacing, *d*=*d*_1_+*d*_2_, and the average refractive index, *n*_av_=0.5(*n*_1_+*n*_2_), which are intrinsically linked to the composition of the BCP. When illuminated at normal incidence, the characteristic wavelength of the photonic multilayer (*λ*_max_) can be estimated from the Bragg equation [Eq. (1)], while the Bragg–Snell equation can be used in more complex cases (Supporting Information, Equation S1). In contrast, the color purity is affected by both intrinsic factors (e.g., the refractive index contrast of the blocks, Δ*n*=|*n*_1_−*n*_2_|) and extrinsic factors, such as the casting or annealing steps that affect the quality, alignment, and size of the lamellae within the overall film. Considering the pairing of PS and P2VP, with refractive indices of *n*_PS_=1.59 and *n*_P2VP_=1.62,^[22]^ it is clear that this system possesses a high average refractive index (*n*_av_=1.61), and a low refractive index contrast (Δ*n*=0.03). The former has the advantage of reducing the domain spacing and consequently the DP needed to produce visible color, although the latter increases the multilayer thickness required for saturated coloration. However, like most LBCP systems, PS‐*b*‐P2VP photonic films do not natively reflect visible light due to insufficient domain spacing in the dry state (*d*<100 nm). As such, methods have been applied to increase *d* and/or *n*_av_ (e.g., homopolymer or nanoparticle doping), thereby extending the working range of PS‐*b*‐P2VP films from the ultraviolet through to the infrared (*λ*_max_≤1821 nm).^[23]^ Alternatively, the characteristic wavelength and color purity can be enhanced by swelling the domains,[^22b^, ^23^, ^24^] either with solvent or by quaternization of the nitrogen in the P2VP block, which allows for selective coordination with anionic salts.^[25]^ However, such strategies often result in transient or unstable coloration.
(1)

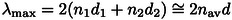




Despite its ubiquity, there are experimental challenges to preparing photonic films from PS‐*b*‐P2VP. To date only five solvents have been reported to form lamellar structures, although given their diversity (i.e., ester, halogenated aliphatic, and aromatic, see Table 1) it would suggest that PS‐*b*‐P2VP should be able to self‐assemble in most polar and aprotic systems. However, given that for all compositions of PS‐*b‐*P2VP the lamellae were only well‐resolved after an extended period of annealing, it also infers that the solvent used is less important relative to the annealing process. Furthermore, while for ultrahigh molecular weight LBCPs a high degree of entanglement inevitably inhibits self‐assembly, the fact that an annealing step is still required for PS‐*b*‐P2VP with a M_n_ of 78 kDa^[22b]^ suggests there is weak microphase separation between these relatively compatible blocks. In contrast, PS‐*b*‐PSM has been reported to form a lamellar structure without annealing for M_n_≤1600 kDa,^[19b]^ allowing for direct access to visibly colored films.

**Table 1:** Linear block‐copolymers used to assemble photonic multilayer films.

Linear block‐copolymer (LBCP)						Self‐assembly	Nanostructure	
Name^[a]^	No. articles	*M*_n_^[b]^ [kDa]	DP_1_^[c]^	DP_2_^[c]^	DP_1_/DP_2_^[c]^	Solvent^[d]^	*d* ^[e]^ [nm]	*λ*_max_^[f]^ [nm]
PS‐*b*‐P2VP	54	78–443	365–2381	380–1855	0.9–1.4	TCE; PGMEA; THF; toluene; 1,4‐dioxane	30–640	UV–1821
PS‐*b*‐PI	15	274–1410	1315–7585	2011–9102	0.4–0.9	DDC; THOH; toluene; DVB; cumene; *o*‐xylene	106–292	305–855
PS‐*b*‐PE/P	4	800–831	3601–3841	5703–6501	0.6–0.7	toluene	≥180	UV–622
PS‐*b*‐P4VP	3	288	2286	471	4.9	chloroform	70–190	350–530
PS‐*b*‐P*t*BMA	2	680	3044	2553	1.2	acrylate monomers; toluene	143–180	428–543
PS‐*b*‐PMAA	1	596	4801	1116	4.3	THF	≥175	520–579
PS‐*b*‐PMMA	1	328	1709	1743	1.0	styrene	120	483
P(E‐*co*‐P)‐ *b*‐P(E‐*co*‐N)	1	576	N/A	N/A	N/A	toluene	90–150	268–448
P(MCP‐*co*‐VTM)‐ *b*‐P(E‐*co*‐N)	1	451	N/A	N/A	N/A	toluene	170	470
LLDPE‐*b*‐ULDPE	1	47–68	N/A	N/A	N/A	N/A	140–205	369–503
PS‐*b*‐PSM	1	700–1600	2880–11 234	1837–2560	1.4–5.6	*o*‐xylene; toluene	178–292	430–625
PTEPM‐*b*‐PSMA	1	139–237	390–666	353–553	1.1–1.2	THF	102–173	UV–591
PI‐*b*‐P4MS	1	821–845	7574–9118	4951–5364	1.5–1.7	chloroform; THF	185–196	≥580
PS‐*b*‐PHEMA	1	216	1455	488	3.0	THF	N/A	N/A
PBD‐*b*‐PEO	1	66	627	731	0.9	THF	78–185	UV–495

[a] For the full names of the LBCPs, refer to Table S1. [b, c] The number average molecular weight (*M*_n_) and the degree of polymerization (DP) for each block were taken directly from the articles. If unavailable, they were calculated from other parameters presented. [d] Solvent is the preparative self‐assembly solvent from which photonic multilayers were fabricated. For the full name of all solvents, refer to the preface of Table S4. [e] *d* is the domain spacing of the formed lamellar structures. If values were not given explicitly, they were extracted from Figures in the article. [f] *λ*_max_ is the characteristic wavelength with the maximum intensity in the spectrum of the photonic multilayers. “UV” indicates that the film reflected within the ultraviolet region and therefore appeared colorless. In all categories, N/A corresponds to no available data in the source article.

### 3.2 New strategies for LBCP photonics

Benchmarking the optical properties of other LBCPs employed for photonics against PS‐*b*‐P2VP reveals that *n*_av_ is generally lower (Table S10), resulting in the need for even thicker domains to achieve visible coloration, and consequently larger MWs or greater swelling. To address this limitation, a number of recent studies have explored the balance between ultrahigh MW and good‐quality self‐assembly for native colored films,[^19b^, ^26^] or the introduction of tunability to the domains via functional chemical groups on the monomers.[^19c^, ^27^] Additionally, several methods (e.g., reversible deactivation radical polymerization) have recently been reported for ultrahigh MW polymers; however, their compatibility with a wide monomer library and applicability for LBCP photonics still needs to be verified.^[28]^ Besides increasing MW, the issue of low *n*_av_ and Δ*n* could be addressed using LBCPs with novel compositions, such as the inclusion of atoms or substituents into the polymer chain with high molar refractivity (e.g., halogens, phosphorus, sulfur, or aromatic rings).^[29]^ Alternatively, nanoparticle doping can be employed to increase *n*_av_ and enhance Δ*n*.^[2a]^ However, as selective doping of the domains is required to optimize the photonic structure, it is also necessary to either identify compatible nanoparticles for specific polymers blocks or introduce compatibilizing surface modification to the nanoparticles.^[30]^ In parallel, the ability of the LBCPs to self‐assemble can be optimized by increasing the incompatibility between the two blocks (i.e., developing high *χ* BCPs).^[31]^

The potential for functionalization is another consideration when the composition of the BCP is chosen. This can range from altering the self‐assembly behavior of the LBCP, as in the case of supramolecular comb‐like BCPs,^[32]^ through to imparting responsivity to the formed lamellar structure. The selectivity and sensitivity to a specific stimulus depends on the chemical structure of the constituent monomers or, more commonly, their potential for further modification. For example, the vinylpyridine (VP) monomer offers many tuning possibilities, of which more than 20 have been reported. These include crosslinking, counterion exchange, applying an electric field, and supramolecular chemistry (Tables S3, S5, and S6), which can control the structural and optical properties of VP‐containing photonic LBCP materials. Such strategies have led to smart photonic materials that are responsive to solvents, humidity, electric field, temperature, and so on. By examining the tuning methods used, it appears that the position of the nitrogen in vinylpyridine determines the properties of the resultant LBCP; in general, P2VP is readily ionized or crosslinked, while P4VP can be complexed with small molecules via supramolecular interactions. However, the versatility of the VP monomer can also be a limitation: a broad responsivity can impede the long‐term color stability when considered as a pigment, or its real‐world viability as a sensor due to poor selectivity. As such, to improve the applicability of BCP photonics as sensors, functionalization approaches with greater specificity are required, while methods to retrospectively tune and lock‐in the color (e.g., crosslinking) can improve their potential as pigments.

### 3.3 The reliance on ROMP for BBCP photonics

BBCPs are increasingly preferred over LBCPs for photonic multilayers due to their more rigid molecular architecture and reduced entanglement during self‐assembly, allowing access to lamellae with larger domain spacings.^[2a]^ The development of photonic BBCP materials is predicated upon the discovery of the Grubbs catalysts and their use in ROMP. Indeed, more than 90 % of the articles in this subcategory employed BBCPs prepared by the *grafting‐through* approach, whereby macromonomers with polymerizable bonds were directly polymerized via ROMP (Tables S6 and S8). Notably, norbornene and its derivatives were selected as the backbone monomer in all these BBCPs. The common use of norbornene‐based macromonomers arises from (i) the ring strain of norbornene (ca. 27.2 kcal mol^−1^),^[33]^ which favors both high ROMP activity and efficient polymerization, while preventing secondary metathesis of the polymer backbone;^[34]^ and (ii) the straightforward functionalization of norbornene with substituents or side chains, which allows for a diverse library of macromonomers to be synthesized and combined to produce novel BBCPs.^[35]^

While ROMP of norbornene‐based monomers has quickly become the dominant method, this polymerization route employs expensive metathesis catalysts, which currently limits the cost‐effective production of BBCPs to laboratory‐scale synthesis (i.e., a few hundred milligrams). In addition, the catalysts required for metathesis typically necessitate rare, and in some cases, “endangered” metal centers.^[36]^ Titanium‐based catalysts are a potential alternative, but their use has been limited by its intolerance to many reactive functional moieties and their protecting groups.^[37]^ Other polymerization methods offer a larger flexibility in terms of catalysts and reaction conditions, including environmentally safer options;^[38]^ however, the synthetic strategy would have to be altered accordingly. For example, two photonic BBCPs have been obtained by a *grafting‐from* approach (whereby monomers were directly polymerized from a LBCP backbone), which was achieved using a combination of reversible ATRP, RAFT, and ring‐opening polymerization (ROP). Although this method is cost‐effective, when compared with ROMP it is a synthetically laborious process involving multiple deprotection and functionalization steps, while also suffering from several issues, including incomplete monomer conversion, low efficiency of polymerization (due to high entanglement), and significant side reactions among the multiple components.[^12^, ^39^] These factors could suggest why further studies on photonic BBCP synthesized using a *grafting‐from* approach have not been reported. In addition, given the increased number of synthetic steps, it is unclear whether the total “environmental cost” is smaller compared to ROMP once total solvent use and additional purification steps are considered. Therefore, while ROMP remains the most viable method to unlock the potential of BBCPs as large‐area colorants (e.g., automotive paint), alternative backbones and polymerization methods are urgently required that can maintain efficient polymerization at high DPs while achieving a lower production cost. Potential alternative polymerization methods that could be applied towards the effective and inexpensive polymerization of BBCPs include metal‐free ROMP,^[40]^ *grafting‐onto* click chemistry,^[41]^ and orthogonal cationic and radical RAFT polymerizations.^[42]^ Finally, recent examples employing supramolecular comb‐like BCPs suggest an alternate strategy that bypasses these synthetic challenges, while also offering a degree of tunability via the formulation.[^32^, ^43^]

## 4 Future Prospects

**Figure 5:**
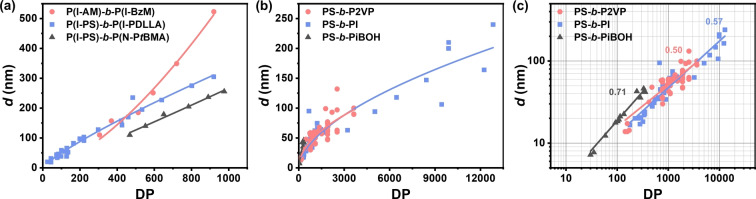
The relationship between domain spacing (*d*) and the degree of polymerization (DP) for selected a) BBCPs and b) LBCPs. This data combines published examples of each specific BCP and is fitted with a power law of the form *y*=*ax* ^*b*^. c) Replotting the LBCP data on logarithmic axes allows for the efficiency of the copolymer system for photonic applications to be quantified. The exponent of the power law (*b*, as stated on the plot) corresponding to the gradient, with a value of 1 corresponding to linear growth. The DP was either taken from the source articles (Supporting Information, Section S4) or, if not directly available, estimated from other reported parameters (e.g., the ratio of the MW of each block with that of the respective monomer). For *d*, where values were not explicitly given, they were extracted from the Figures in the source article.

To move towards rational polymer design, beyond optimizing the optical properties of the monomers, it will be necessary to understand how efficiently the domain spacing, *d*, increases with the DP of the BCP. This issue is especially important as we seek to improve the atom and material economy of these photonic materials as they are increasingly considered for large‐area coloration. While the drying conditions can affect the quality of the lamellae (in terms of alignment, size, etc.), the packing behavior of the individual BCPs is typically intrinsic to the monomers used in each block. For example, BBCPs are typically believed to pack in an extended conformation, giving rise to pseudolinear relationships between *d* and DP (and even the ability to linearly blend BBCPs of significantly different DP),^[14]^ which enables the easy production and control of structural colors from BBCPs (Figure 5a).^[44]^ Conversely, for LBCPs, more compact conformations result in nonlinear relationships, with an increase in DP not coherently translating into an equivalent increase in *d* and thus *λ*_max_ (Figure 5b). Historically, there has been a race to produce BCPs with larger and larger MWs in an attempt to directly reach photonic dimensions; however, with conformational issues becoming increasingly prevalent at ultrahigh MWs, the additional synthetic effort typically yields diminishing returns in terms of *d*. A more effective strategy would be to find monomers with less compact conformations, which could lead to direct access to LBCP‐based systems with native structural coloration. The importance of different LBCP conformations can be appreciated by replotting the relationship between *d* and DP with logarithmic axes, as shown in Figure 5c. How effectively an increase in DP translates into a larger domain size can be inferred from the gradient of the plots, with a steeper gradient (approaching 1) indicative of a more linear relationship and thus more likely to achieve reflection at larger wavelengths. However, within just these three PS‐based LBCPs there is significant variance in the gradient, making it difficult to speculate on the chemical or structural parameters that affect the self‐assembly performance of each monomer and/or copolymer pair. A broader meta‐analysis of known BCP systems could reveal the criteria determining the relationship between *d* and DP, and through that, propose and assess candidates for ideal monomers *in silico*.

This meta‐analysis could be combined with molecular dynamics simulation to reveal information about the importance of individual monomer and synthetic choices beyond simple polymeric (e.g., DP) or optical parameters (e.g., *n*). For example, how non‐covalent bonding motifs or the chain conformation in solution influence the formation and packing of the lamellae, or how the thickness of the boundary layer affects the domain spacing. Furthermore, analysis of the surface composition of a densely grafted BBCP may reveal potential opportunities for further functionalization. Specifically, if the end groups of the MM are exposed, they might play a disproportionate role in the overall phase behavior. Given that the end groups typically correspond to the initiator moiety, they could potentially be used as a handle to functionalize the “surface” and, as such, tailor the self‐assembly process.

Alternatively, by exploiting new nanoarchitectures, the dimensionality of the polymers can be decoupled from the photonic response, which avoids the synthetic challenges and conformational issues associated with a requirement for ultrahigh MW polymers. While this is not possible in the context of lamellar structures, where the domain spacing correlates with the BCP dimensions, the recent discovery of photonic BBCP particles based on porous nanostructures with short‐range order (i.e., an inverse photonic glass,^[16]^ Figure 4c) offers a potential solution. For these particles, the optical response is governed by the center‐to‐center distance of the correlated pores and the refractive index contrast between the BBCP and the pore media (e.g., air, solvent). As a result, the size of the polymer is no longer determinant, allowing for the use of BBCPs with much smaller DPs (i.e., DP>50 vs DP>250 for lamellae), and with similar refractive indices, allowing for a wider monomer choice and easier access to higher *n*_av_. In addition, while studies into lamellar systems emphasize the requirement for equal DP ratios to achieve a planar interface, the potential to create photonic structures using swollen micelles as a template brings a new parameter space, with new requirements (e.g., amphiphilicity). Furthermore, given that the reflected color of such particles depends less on the intrinsic properties of the BBCP and more upon the fabrication conditions (e.g., emulsification parameters and drying conditions^[3b]^), these systems are inherently tunable, which allows for a spectrum of colors to be prepared from a single DP.^[3b]^ As such, this discovery permits a radical reconsideration of the optical and chemical properties required for BCP structural color and unlocks new avenues of research. However, it is also important to note that the precise formation mechanism of such photonic porous particles is not yet fully understood and that they have not been replicated at photonic dimensions with LBCPs, suggesting that the role of amphiphilic BBCPs as “giant surfactants” needs to be investigated further.^[45]^

Finally, the ability to access a larger pool of available BCPs is crucial in terms of solving the often‐neglected issue of the biocompatibility and biodegradability of the final photonic material. As shown in Tables S5–S9, the BCPs currently used to produce photonics are typically highly synthetic and potentially do not degrade within the natural environment. The widespread release of polymer waste (e.g., microplastics) into natural ecosystems is threatening the health and habitat of many living species and also potentially causing long‐term problems for humanity.^[46]^ Therefore, it is ethical for the community to consider biocompatibility and/or biodegradability when promoting new polymer systems for photonics and, in particular, pigments. The incorporation of degradable and biocompatible elements into the main chains of LBCPs or the backbones and sidechains of BBCPs offers an immediate solution; however, a truly sustainable polymer system necessitates the use of biopolymers (e.g., polylactic acid, polycaprolactone, polysaccharides, and polypeptides).

Finally, by employing green chemistry approaches to both the synthesis (e.g., catalysts, solvents) and fabrication steps (e.g., film casting or micro‐emulsification), it is possible to reduce the environmental impact of the overall process. For example, recent studies have reported that LBCPs can be produced via a mild, one‐pot polymerization method,^[47]^ which could reduce energy costs and material waste. Alternatively, the use of harmful organic solvents can be avoided by switching to aqueous polymerization techniques.[^38^, ^48^]

## 5 Summary

Over the last two decades BCPs have been successfully used to prepare photonic structures via a self‐assembly approach. While earlier examples focused on LBCPs, BBCPs have become increasingly dominant as they offer the accessible ultrahigh MWs and robust self‐assembly required for native photonic structures. Additionally, significant effort has been applied to understanding how physical methods such as swelling, doping, and external stimuli can be applied to change the structural parameters of the self‐assembled structures, thus tuning the optical response of the obtained photonic materials. Finally, the soft confinement of the self‐assembly process within an emulsified droplet has recently gained traction as a scalable method to produce hierarchical particles, with potential application as pigments.

For LBCPs, a historical preference for anionic polymerization combined with practical limitations in assembling photonic films with sufficiently large domain spacing (e.g., MW, entanglement, annealing) has resulted in PS‐*b*‐P2VP continuing to dominate. This is compounded with the increasing use of commercial polymers, limiting the impetus for new LBCP systems to be developed. While side‐chain diversity is much greater for BBCPs, the ubiquity of ROMP has resulted in a near universal use of norbornene‐based backbones. To catalyze the discovery of new architectures and applications, it is now necessary to widen the range of materials employed. In order to obtain photonic materials effectively, new compositions of BCPs are required to achieve a rapid increase of domain size with the degree of polymerization, while maintaining a high refractive index contrast. Alternatively, new architectures can allow the optical and polymer properties to be decoupled, offering new parameter spaces to be explored. In addition, a consideration of the scalability of the synthetic pathway from a process chemistry viewpoint is increasingly necessary as the community moves from fundamental research to applications. This is especially true for BBCPs, where metathesis polymerization continues to be the dominant strategy. Finally, to reduce the threat to the environment, biocompatible and biodegradable BCPs are crucial if these photonic materials are to be ethically transferred from that of a laboratory‐scale proof‐of‐concept to industrial‐scale colorants.

## Disclaimer

The opinions expressed in this publication are the view of the author(s) and do not necessarily reflect the opinions or views of *Angewandte Chemie International Edition*/*Angewandte Chemie*, the Publisher, the GDCh, or the affiliated editors.

## Conflict of interest

The authors declare no conflict of interest.

6

## Biographical Information

*Zhen Wang received his B.Sc. and M.Sc. in chemistry from Renmin University of China. Currently, he is pursuing a Ph.D. under the supervision of Prof. Silvia Vignolini, in the Bio‐inspired Photonics group within the Yusuf Hamied Department of Chemistry at the University of Cambridge. His research interests focus on the synthesis of bio‐compatible brush block‐copolymers and their applications towards structural coloration*.

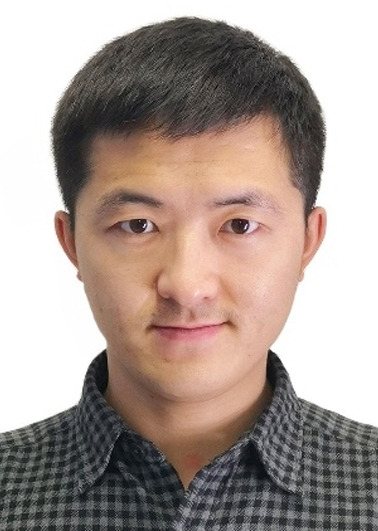

## Biographical Information

*Richard M. Parker graduated from the University of Southampton (UK) with a degree in chemistry (M.Chem.) in 2007, followed by the award of a Ph.D. in chemistry with optoelectronics in 2011. In 2012, he moved to the University of Cambridge (UK), where he is currently a Senior Research Associate. His research exploits self‐assembly processes within ink‐jet or microfluidic droplets to prepare novel material architectures, ranging from supramolecular microcapsules and gels, to photonic microparticles and structurally colored films*.

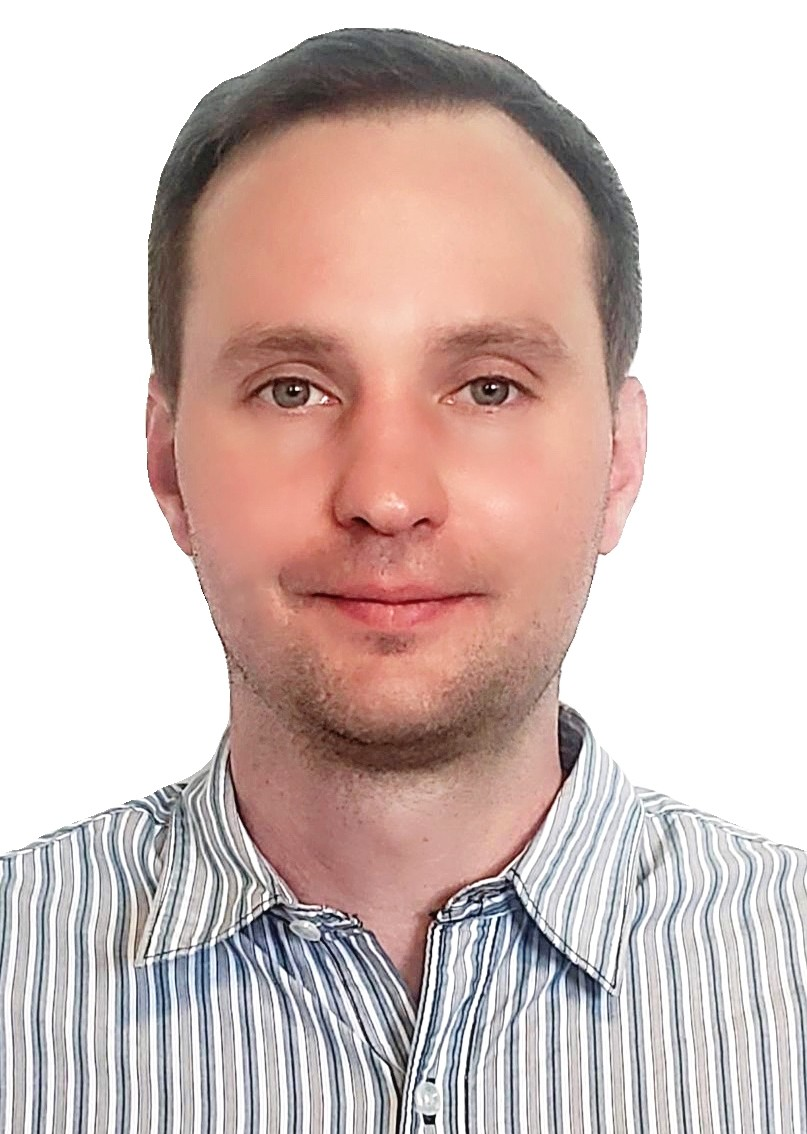

## Biographical Information

*Chun Lam Clement Chan graduated in 2017 with a degree in natural sciences (M.Sc.) from the University of Cambridge (UK), where he recently completed a Ph.D. under the supervision of Prof. Silvia Vignolini at the Yusuf Hamied Department of Chemistry. His research focuses on developing solid‐state photonic structures based on cellulosic liquid crystal polymers and other biocompatible polymeric systems*.

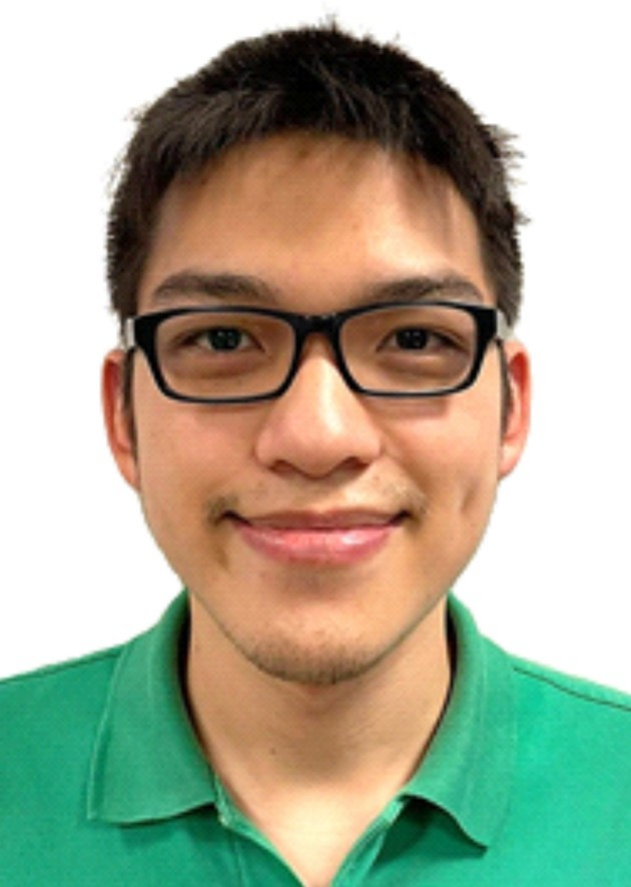

## Biographical Information

*Silvia Vignolini studied physics at the University of Florence (Italy). In 2009, she was awarded a Ph.D. in solid‐state physics at the University of Florence. In 2010, she moved to Cambridge as a post‐doctoral research associate working in the Cavendish Laboratory and the Plant Science Department. She started her independent research by becoming a BBSRC David Philip Fellow in 2013. She is currently a Professor of Chemistry and Bio‐materials at the University of Cambridge*.

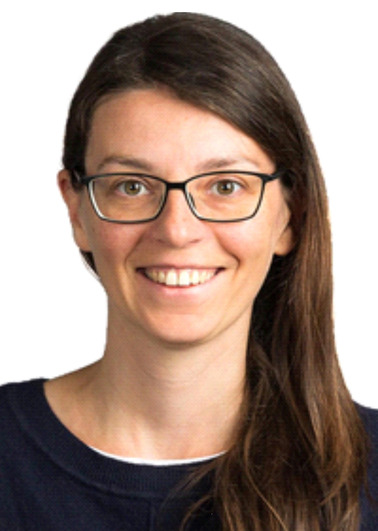

## Supporting information

As a service to our authors and readers, this journal provides supporting information supplied by the authors. Such materials are peer reviewed and may be re‐organized for online delivery, but are not copy‐edited or typeset. Technical support issues arising from supporting information (other than missing files) should be addressed to the authors.

Supporting Information

## Data Availability

The data that supports the findings of this study are available in the supplementary material of this article.
